# Characteristics of Brain White Matter Microstructure in HIV Male Patients With Primary Syphilis Co-Infection

**DOI:** 10.3389/fneur.2021.776818

**Published:** 2022-01-18

**Authors:** Yu Qi, Rui-Li Li, Yuan-Yuan Wang, Wei Wang, Xu-Ze Liu, Jing Liu, Xing Li, Xiao-Dong Zhang, Wen Yu, Jiao-Jiao Liu, Yi-Fan Guo, Bo Rao, Hong-Jun Li

**Affiliations:** ^1^Department of Radiology, Beijing Youan Hospital, Capital Medical University, Beijing, China; ^2^Department of Radiology, The Second Hospital of Beijing, Beijing, China; ^3^School of Computer Science and Engineering, Northeastern University, Shenyang, China; ^4^Department of Radiology, The Affiliated Infectious Diseases Hospital of Soochow University, Suzhou, China; ^5^Department of Radiology, Tianjin First Central Hospital, School of Medicine, Nankai University, Tianjin, China; ^6^Geriatric Department, Zhejiang Provincial People's Hospital, People's Hospital of Hangzhou Medical College, Hangzhou, China; ^7^Department of Radiology, The First Affiliated Hospital of Zhejiang Chinese Medical University (Zhejiang Provincial Hospital of Traditional Chinese Medicine), Hangzhou, China; ^8^Department of Radiology, Zhongnan Hospital of Wuhan University, Wuhan University, Wuhan, China

**Keywords:** HIV, syphilis, white matter, diffusion tensor imaging, tract-based spatial statistics

## Abstract

**Purpose:** To investigate the effect of syphilis infection on the microstructure of white matter (WM) in HIV-infected male patients using diffusion tensor imaging (DTI).

**Methods:** Twenty-seven HIV-infected male patients with current syphilis or a history of syphilis (HIV +/syphilis +), twenty-nine HIV-infected male patients without syphilis co-infection (HIV +/syphilis–), and twenty-nine healthy controls (HC) were enrolled. All participants received DTI, and all patients received comprehensive neuropsychological assessment. Tract-based spatial statistics (TBSS) was adopted to analyze the DTI measures: fractional anisotropy (FA), mean diffusivity (MD), axial diffusivity (AD), and radial diffusivity (RD). Correlation analysis was conducted to investigate the relationships between DTI measures and cognitive performance.

**Results:** There were no significant differences in DTI measures between HIV+/syphilis– and HC. Compared with HC, lower FA was found in body of corpus callosum (BCC), splenium of corpus callosum (SCC), genu of corpus callosum (GCC), the bilateral anterior corona radiata (ACR), superior corona radiata (SCR), posterior corona radiata (PCR), and posterior thalamic radiation (PTR) in HIV+/syphilis+ (*p* < 0.05). Higher RD was found in BCC and SCC (*p* < 0.05). Compared with HIV+/syphilis–, lower scores were found in complex motor skills (CMS) in HIV+/syphilis+, lower FA was found in BCC, SCC, GCC, the bilateral ACR, SCR, PCR, PTR, cingulate gyrus (CGC), the right inferior fronto-occipital fasciculus (IFO), the retrolenticular part of internal capsule (RLIC), sagittal stratum (SS), external capsule (EC) in HIV+/syphilis+ (*p* < 0.01). Correlation analysis uncorrected for multiple comparisons showed there was a positive correlation between FA in GCC and CMS, FA in BCC, and CMS in HIV+/syphilis+.

**Conclusions:** Syphilis co-infection can have an additive or synergistic effect on the brain WM in HIV-infected subjects. HIV-infected patients without syphilis should be actively treated to avoid syphilis infection.

## Introduction

Human immunodeficiency virus (HIV) can penetrate into the central nervous system (CNS) during the early stage of infection. One study showed that viral RNA could be detected in the cerebrospinal fluid (CSF) within as early as 8 days of HIV infection (1). The astrocytes, microglia, oligodendrocytes, and other cells can be destroyed by the virus after penetration (2), resulting in changes in the microstructure of white matter (WM) (2, 3). The incidence of syphilis in some European regions has increased in recent years, which mainly occurs in men who have sex with men (MSM) (4, 5). Syphilis infection caused by the spirochaete bacterium Treponema pallidum has the same group of patients with HIV; the incidence of syphilis is 77 times greater in HIV-infected patients than in the general population (5, 6). Syphilis infection involves primary syphilis, secondary syphilis, and tertiary syphilis. About 25–40% of infected patients can have “neuroivasion” during infection, especially in the stage of primary syphilis and secondary syphilis. Due to the CSF can clear the infection automatically, people in the stage of primary syphilis and secondary syphilis may not have any CNS symptoms. When CSF fails to clear the infection, neurosyphilis develops (7).

Cooperative relationship between syphilis and HIV can be found. Syphilis had a negative impact on the CD4+T cell counts in HIV-positive patients regardless of treatment (8). HIV-infected patients with CD4+T counts <350 μl/L were more likely to acquire neurosyphilis (9). When immune system is seriously impaired, neurosyphilis tends to progress (10). Syphilis co-infection may cause neurocognitive impairment. HIV patients with prior syphilis co-infection had a greater number of impaired neuropsychological (NP) test domains and more impaired in the NP learning domain (11). HIV-infected subjects with a history of syphilis had a poor performance in the cognitive domains of memory and learning (12). The mechanism of cognitive impairment caused by syphilis and HIV co-infection remains unclear; one possible reason is that Treponema pallidum may be present in CSF at some point during syphilis infection, although it did not develop into neurosyphilis. Treponema pallidum may increase HIV viral load in CSF of patients with syphilis and HIV co-infection, resulting in impaired neurocognitive function (10, 13).

The damage of microstructure in WM can be detected by diffusion tensor imaging (DTI). Unlike gray matter and CSF, myelinated axon of WM consists of myelin, axonal membrane, microtubules, and neurofilaments, which could hinder water diffusion and cause the perpendicular diffusion coefficient smaller than the parallel diffusion coefficient (14). When pathological changes occur in WM, the diffusion rate in parallel and perpendicular directions decreased (15, 16). Among various DTI-derived scalar measures, we investigated fractional anisotropy (FA), mean diffusivity (MD), axial diffusivity (AD), and radial diffusivity (RD) since these measures have been widely used as indices to quantify brain injury in people with HIV infection. FA (the ratio of anisotropic to isotropic diffusion) provides information about the integrity of the WM, especially the integrity of membranes (14), but it is not specific to the type of injury. MD can reflect the magnitude of water diffusion, but not the direction. AD reflects the diffusion rate in parallel direction; conditions of axonal damage such as microtubules depolymerized and fast axonal transport inhibited can decrease AD by up to 30–50% (16). RD reflects the diffusion rate in the perpendicular direction. Myelin loss can lead to increased apparent dispersion coefficient (ADC) by 75% (16).

Several studies have investigated the relationship between the damage to WM and cognitive impairment in HIV-infected patients. Chang et al. (17) found that, for patients with antiretroviral-stable HIV, the decline in the global deficit score was related to the increased MD in the genu of callosum and decreased FA in the parietal and frontal WM and putamen. Cysique et al. (18) studied the HIV-infected patients who were clinically stable with successful viral control and showed that, compared with both the control group and patients with asymptomatic neurocognitive impairment (ANI), the patients with mild neurocognitive disorder (MND) or HIV-associated dementia (HAD) had lower FA values in cingulum and fornix, higher MD values in the fornix and external capsule. Li et al. (19) found increased AD and MD in periventricular WM in patients with untreated ANI through comparing them with normal controls; the result suggested that the cause of microstructure damage in patients with untreated HIV with ANI was axonal injury rather than demyelination.

However, there was no study on the characteristics of microstructure changes in WM of patients with syphilis and HIV co-infection and the cognitive status at present. Males are the predominantly affected population of syphilis and HIV co-infection (5), so, in order to understand the effect of primary syphilis infection on the microstructure of WM in HIV-infected male patients, we restricted our sample to only males. We investigated WM through comparing the differences of WM among healthy controls (HC), HIV-infected male patients with and without syphilitic infection. This study may help us understand the mechanism of cognitive decline in patients with HIV and primary syphilis co-infection.

## Methods

### Participant Selection

Between June 2015 and June 2017, twenty-seven HIV-infected male patients with current syphilis or a history of syphilis (HIV+/syphilis+) and twenty-nine HIV-infected male patients without syphilis co-infection history (HIV+/syphilis–) were enrolled in this study. Twenty-nine seronegative HC matched for age, gender, and education level were recruited from the same community by advertisements. This study was conducted in accordance with the guidelines of the Declaration of Helsinki and was approved by the ethics committee of Beijing Youan Hospital, Capital Medical University, and informed consent was obtained from all the patients or their relatives or guardians. The inclusion criteria were as follows: (1) the patients with HIV were diagnosed by an enzyme-linked immunosorbent assay and western blot analysis; (2) the patients received T pallidum particle agglutination (TP-PA) and the Rapid Plasma Reagin (RPR); (3) syphilis infection occurred after HIV infection; (4) male; (5) age had to be from 18 to 50 years; (6) the patients underwent a comprehensive neuropsychological assessment and had complete clinical and imaging data. The exclusion criteria were as follows: (1) patients with HIV/AIDS transmitted by drug abuse or mother-to-child vertical transmission; (2) anxiety, depression, alcoholism, metabolic encephalopathy, vitamin B12 deficiency, and drug interaction; (3) central nervous system diseases: tumors, cerebrovascular diseases, opportunistic infections, such as cryptococcus, toxoplasma, cytomegalovirus encephalitis, progressive multifocal leukoencephalopathy (PML), etc., other visible diseases on MRI [T1WI and T2-fluid attenuated inversion recovery (FLAIR)]; (4) the patients with MR contraindications: pacemakers, defibrillators, implanted electronic systems, vascular clips, mechanical heart valves or cochlear implants; (5) the patients with a sudden onset of illness.

To assess the cognitive status, all patients underwent a comprehensive neuropsychological assessment, which included six cognitive domains (3). The neurocognitive evaluation surveys contained complex motor skills (Grooved Pegboard Test), speed of information processing (Trail Marking Test A, TMT-A), memory (Hopkins Verbal Learning Test, HVLT-R; Brief Visuospatial Memory Test, BVMT-R), executive function (Wisconsin Card Sorting Tests, WCST-64), attention and working memory (Continuous Performance Test-Identical Pair, CPT-IP; Wechsler Memory Scale, WMS-III; Paced Auditory Serial Addition Test, PASAT), and verbal fluency (Animal Verbal Fluency Test, AFT). Raw scores for each test are converted to T-scores, which are adjusted for age and education level. T-scores on over one test for one cognitive domain were averaged to get mean T-scores.

Clinical and demographic data, including age, duration of infection, highly active antiretroviral therapy (HAART) medication history plasma CD4 count, and the CD4/CD8 ratios and plasma HIV viral load, were collected. The duration of HIV infection was described by the patient's own account. The recent CD4+ counts and the CD4/CD8 ratios were collected within 2 weeks of the MRI. Target not detected (TND): HIV viral load is <40 copies/ml.

All HIV-infected patients received TP-PA and RPR to diagnose the current status of syphilis infection or a history of syphilis. All the patents with current syphilis infection or a history of syphilis were administered with penicillin during diagnosis.

### MRI Data Acquisition

All the patients received MRI scanning using a Siemens Trio 3.0 Tesla scanner. Standard structural imaging was collected using axial T1WI and T2-FLAIR-combined fat saturation. DTI data were acquired using a single-shot echo planar imaging sequence. Scanning parameters for T2-FLAIR-combined fat saturation: TR = 8,000 ms, TE = 97 ms, inversion time = 2,370.9 ms, phase-encoding directions were AP. Scanning parameters for T1WI: TR = 250 ms, TE = 2.46 ms, flip angle = 9°, field of vision = 256 × 224, acquisition matrix = 256 × 256, section thickness = 1 mm, section number = 176, phase-encoding directions were AP. Scanning parameters for DTI: TR = 3,300 ms, TE = 90 ms, slice thickness = 4 mm with a 1.2 mm gap, number of slices = 63, matrix size = 128 × 128, field of view = 230 × 230, number of excitations = 3, space resolution = 1.8 × 1.8 × 4 mm, total acquisition time = 3.39 min, phase-encoding directions were AP. Diffusion-sensitizing gradients were applied along 20 non-collinear directions with b = 1,000 s/mm2, and b = 0 s/mm2; one b0 was acquired.

### MRI Data Processing

DTI data were processed using FSL5.0.9 (http://neuro.debian.net/). There were three steps for data preprocessing. Firstly, eddy current correction with FDT diffusion module was adopted to eliminate head deformation caused by head movement and eddy current during scanning. Secondly, brain mask image was performed on no-diffusion weighting (b = 0) using the Brain Extraction Tool in FSL (20). Finally, the tensor was calculated using the function of DTIFIT with the FDT diffusion module in FSL, and the four measures: FA, MD, AD, and RD were obtained. There were four steps for tract-based spatial statistics (TBSS) processing using FSL5.0.9. Firstly, all FA data of subjects were aligned with a common space using the non-linear registration tool FNIRT (21, 22), which uses a b-spline representation of the registration warp field (23). Secondly, the mean of all FA images was skeletonized to generate a mean FA skeleton image (threshold = 0.2) to isolate voxels containing gray matter and cerebrospinal fluid in subsequent processing. Then, the skeletonized FA map of all subjects was constructed by projecting all FA data onto the mean FA skeleton image. The skeletonized maps of other measures (MD, AD, and RD) were obtained similarly. Finally, the skeleton-based statistical analysis was conducted for the four measures.

### Voxel-Wise Group Comparisons of DTI Parameters Using TBSS Analysis

For TBSS analysis, voxel-wise statistics was carried out in the general linear model (GLM) module using FSL randomize tool with a non-parametric permutation testing (5,000 random permutations). The threshold-free cluster enhancement (TFCE) approach was adopted for multiple comparison corrections. All the results were considered significant with a family-wise error (FWE) correction at the level *p* < 0.05. Age and education were considered as covariates in this study. For each of the measures (FA, MD, AD, and RD), F-Test was used to compare the three groups (HC, HIV+/syphilis– and HIV+/syphilis+), and then individual *t*-test was used to compare two-group difference when an F-test was significant. The Johns Hopkins University (JHU)-ICBM-DTI-81 WM Label Atlas was adopted to identify the differences in white matter fibers (24).

### Region of Interest-Based Analysis of DTI Measures

The group comparison showed different clusters between HC and HIV+/syphilis+, and between HIV+/syphilis+ and HIV+/syphilis–. Therefore, we performed ROI-based analysis to define group differences between HC and HIV+/syphilis+ and HIV+/syphilis+ and HIV+/syphilis–. We subdivided different clusters into different WM regions by JHU-ICBM-DTI-81 WM Label Atlas, and then we extracted the mean DTI measures of each participant from each different cluster in different WM regions and made a comparison between groups.

### Statistical Analysis

Analysis was conducted with IBM SPSS Statistics 25.0. *p* < 0.05 was considered statistically significant. All normally distributed variables were reported as mean ± SD, while non-normally distributed variables were reported as median (25th−75th percentile). ANOVA and *t*-test were conducted for normally distributed data, and Kruskal–Wallis test and Mann–Whitney *U*-Test were conducted for non-normally distributed data. Chi-square Test was conducted for categorical data. Pearson's correlation analysis was employed to assess the relationship between DTI measures and cognitive performance in HIV+/syphilis+; Bonferroni correction was used after correlation analysis for multiple comparisons.

## Results

### Clinical and Demographic Data

This study enrolled 27 HIV+/syphilis+ male patients, 29 male patients with HIV+/syphilis– and 29 healthy controls. The clinical and demographic data are listed in Table 1. In our study, there were two kinds of patients in the HIV+/syphilis+ group: 11 patients had a history of primary syphilis, and 16 patients were diagnosed as primary syphilis. No significant differences were found in age and education among the three groups (*p* > 0.05). There were no significant differences in CD4 count, CD4/CD8 ratios, duration of infection, treatment conditions or ratio of target not detected (TND) of HIV viral load between HIV+/syphilis+ and HIV+/syphilis–.

**Table 1:** Clinical, demographic data and neuropsychological data of the three groups.

**Items**	**Healthy Controls (29)**	**HIV+/syphilis+ (27)**	**HIV+/syphilis−(29)**	***P*-value**
Age (years) (Mean ± SD)	32.3 ± 4.270	32.89 ± 7.149	30.52 ± 4.771	0.289^a^
Education (year), median (IQR)	16 (16, 16)	16 (16, 16)	16 (16, 17)	0.115^a^
CD4 (cells/ml) (mean ± SD)	/	506.40 ± 185.925	486.07 ± 183.638	0.683^b^
CD4/CD8, median(IQR)	/	0.49 (0.35, 0.72)	0.55 (0.43, 0.63)	0.799^c^
HIV infection (months), median (IQR)	/	20 (9, 41)	21 (16, 36)	0.806^c^
Untreated	/	8/27	6/29	0.539^d^
Ratio of TND of HIV viral load	/	21/27	19/29	0.223^d^
VF, median(IQR)	/	44 (38, 55)	47 (39, 52)	0.825^c^
A/WM (Mean ± SD)	/	42.43 ± 6.500	41.76 ± 5.290	0.675^b^
EF, median (IQR)	/	53.5 (45.3, 60.5)	55 (50.8, 64.4)	0.337^a^
Memory (LDR) (mean ± SD)	/	43.04 ± 6.876	45.11 ± 7.610	0.290^b^
SIP (mean ± SD)	/	42.15 ± 8.654	45.83 ± 8.251	0.110^b^
CMS (mean ± SD)	/	40.30 ± 10.433	47.22 ± 8.519	0.009^b^

*HIV+/Syphilis+, HIV-infected patients with current syphilis or a history of syphilis; HIV+/syphilis–, HIV-infected patients without syphilis co-infection history; TND, target not detected, HIV viral load <40 copies/ml; VF, verbal fluency; A/WM, attention/working memory; EF, executive function; SIP, speed of information processing; LDR, learning/delayed recall; CMS, complex motor skills*.

a *Kruskal–Wallis test*.

b *Independent t-test*.

c *Mann–Whitney U-Test*.

d *Chi-square Test; significance level, p < 0.05*.

### Neuropsychological Data Between the Group of HIV+/Syphilis+ and HIV+/Syphilis–

Table 1 shows the differences in neuropsychological assessment between the group of HIV+/syphilis+ and HIV+/syphilis–. There were no significant differences in verbal fluency (VF), attention/working memory (A/WM), executive function (EF), memory (learning/delayed recall, LDR), and speed of information processing (SIP) between the two groups. But compared with HIV+/syphilis–, the scores in complex motor skills (CMS) were significantly lower in HIV+/syphilis+ (*p* = 0.009).

### White Matter Integrity Evaluation

#### ANOVA Among Healthy Controls, HIV+/Syphilis– and HIV+/Syphilis+

**Figure 1:**
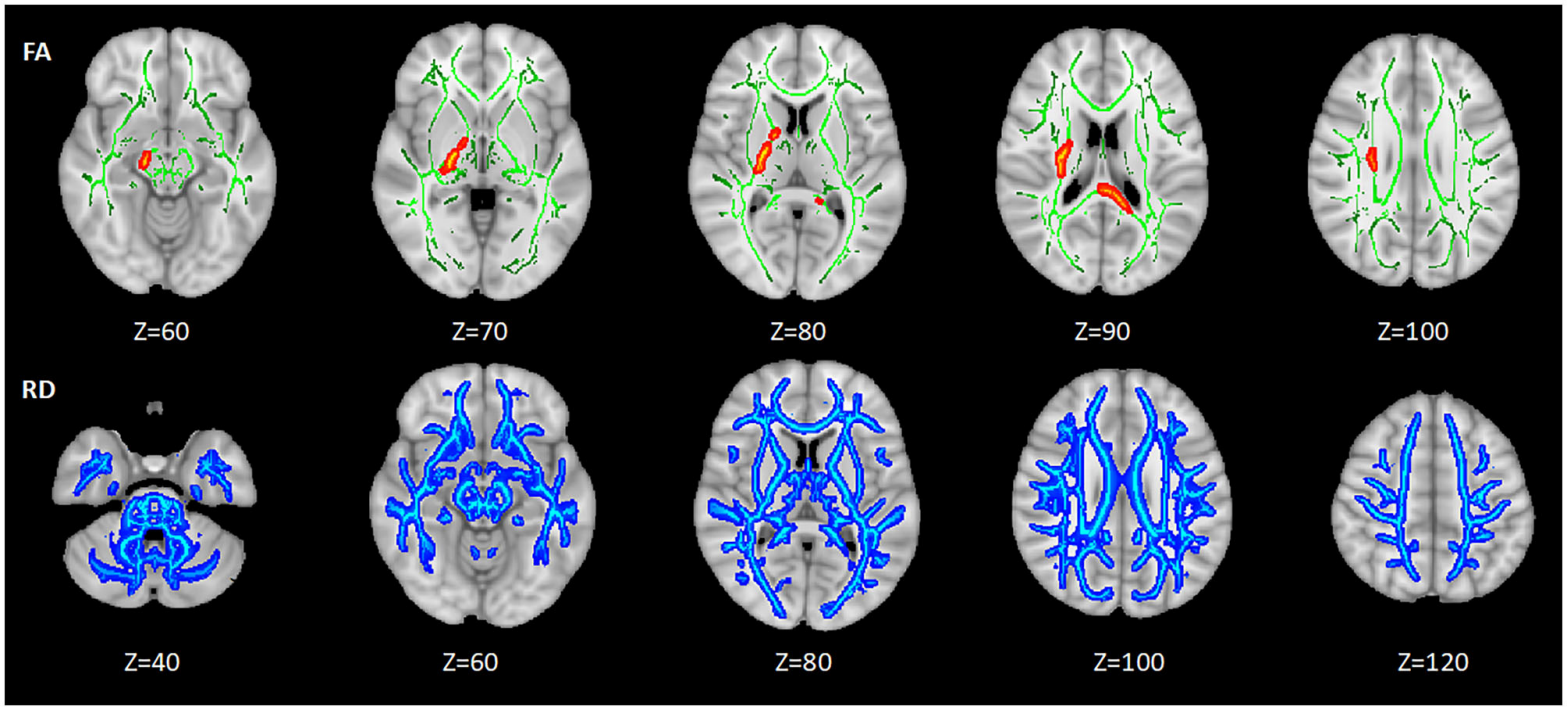
The differences of DTI measures among the three groups. Areas in red and blue represent brain regions with significant difference in FA and RD (FWE-corrected *p* < 0.05). Green represents mean white matter skeleton of all subjects. The number below each brain image indicates the Z coordinate in the Montreal Neurological Institute (MNI) space.

Among the three groups, the abnormal areas in FA mainly concentrated around the lateral ventricle, mainly including the portion of the corpus callosum (CC), internal capsule (IC), superior corona radiata (SCR), etc. The abnormal areas in RD were broad, which nearly involved the whole brain. There were no significant differences in MD and AD of WM among HC, HIV+/syphilis– and HIV+/syphilis+ (Figure 1).

#### HIV+/Syphilis– vs. Healthy Controls

There were no significant differences in FA and RD of WM between patients with HIV+/syphilis– and HC.

#### HIV+/Syphilis+ vs. Healthy Controls

**Figure 2:**
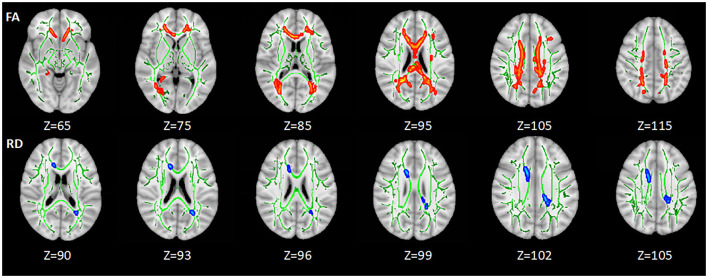
The differences of DTI measures between healthy controls and HIV+/syphilis+ group. Areas in red represent brain regions with significant lower FA in the HIV+/Syphilis+ group compared with healthy controls. Areas in blue represent brain regions with significant higher RD in the HIV+/Syphilis+ group compared with healthy controls (FWE-corrected *p* < 0.05). Green represents mean white matter skeleton of all subjects. The number below each brain image indicates the Z coordinate in the MNI space.

Compared with HC, lower FA was found in the body of corpus callosum (BCC), splenium of corpus callosum (SCC), genu of corpus callosum (GCC), the bilateral anterior corona radiata (ACR), SCR, posterior corona radiata (PCR), and posterior thalamic radiation (PTR) in HIV+/syphilis+ (*p* < 0.05). Higher RD was found in BCC and SCC (*p* < 0.05) (Figure 2; Table 2).

**Table 2:** Comparison of DTI measures between healthy controls and HIV+/syphilis+ in ROI-based analysis.

**DTI measures in ROI**	**Healthy controls**	**HIV+/syphilis+**	***P*-value**
**FA**			
BCC	0.55877 ± 0.04326	0.51681 ± 0.04464	0.001
SCC	0.62763 ± 0.02675	0.59781 ± 0.03843	0.001
GCC	0.63199 ± 0.03200	0.60457 ± 0.03018	0.002
ACR-L	0.45482 ± 0.03589	0.42847 ± 0.03186	0.005
ACR-R	0.46536 ± 0.03514	0.42824 ± 0.03890	<0.001
PCR-L	0.42706 ± 0.03191	0.39951 ± 0.03013	0.002
PCR-R	0.43218 ± 0.02067	0.40803 ± 0.02782	<0.001
SCR-L	0.49114 ± 0.04256	0.46664 ± 0.02969	0.016
SCR-R	0.48842 ± 0.03951	0.46793 ± 0.03304	0.041
PTR-L	0.55323 ± 0.04349	0.51937 ± 0.04662	0.007
PTR-R	0.56831 ± 0.03672	0.53621 ± 0.04037	0.003
**RD**			
BCC	0.000481 ± 0.000058	0.000538 ± 0.000051	<0.001
SCC	0.000496 ± 0.000035	0.000542 ± 0.000046	<0.001

*BCC, body of corpus callosum; SCC, splenium of corpus callosum; GCC, genu of corpus callosum; ACR, anterior corona radiata; PCR, posterior corona radiata; SCR, superior corona radiata; PTR, posterior thalamic radiation; FA, fractional anisotropy; RD, radial diffusivity; L, left; R, right*.

#### HIV+/Syphilis+ vs. HIV+/Syphilis–

**Figure 3:**
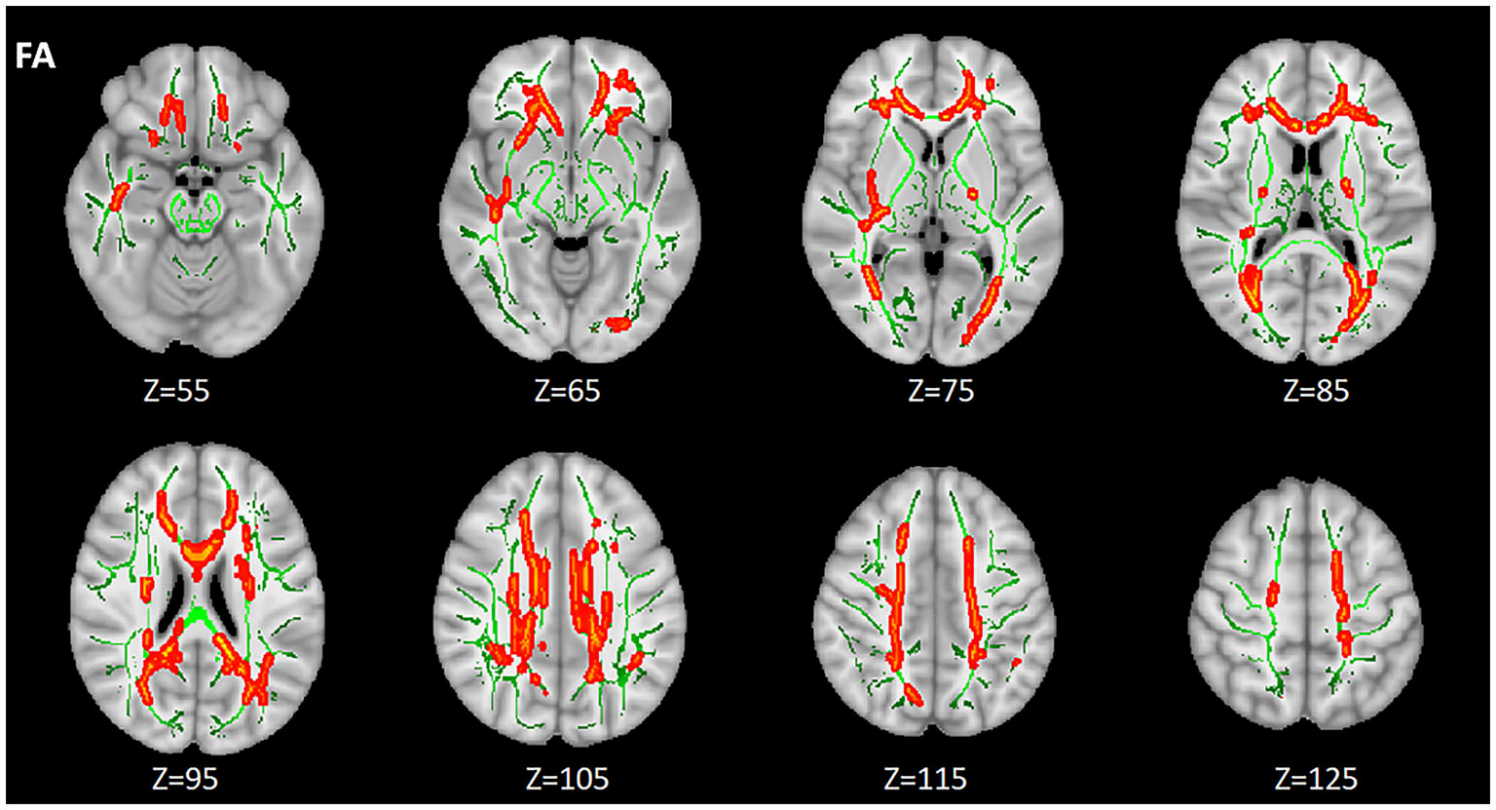
The differences of FA between the HIV+/syphilis– group and the HIV+/syphilis+ group. Areas in red represent brain regions with significant lower FA in the HIV+/Syphilis+ group compared with the HIV+/Syphilis– group (FWE-corrected *p* < 0.01). Green represents mean white matter skeleton of all subjects. The number below each brain image indicates the Z coordinate in the MNI space.

Compared with HIV+/syphilis–, lower FA was found in BCC, SCC, GCC, the bilateral ACR, SCR, PCR, PTR, cingulate gyrus (CGC), the right inferior fronto-occipital fasciculus (IFO), retrolenticular part of internal capsule (RLIC), sagittal stratum (SS), external capsule (EC) in HIV+/syphilis+ (*p* < 0.01) (Figure 3; Table 3).

**Table 3:** Comparison of the FA measure between HIV+/syphilis– and HIV+/syphilis+ in ROI-based analysis.

**FA measures in ROI**	**HIV+/syphilis−**	**HIV+/syphilis+**	***P*-value**
GCC	0.64684 ± 0.33640	0.61718 ± 0.02734	0.001
BCC	0.56280 ± 0.03877	0.52484 ± 0.04363	0.001
SCC	0.60810 ± 0.03056	0.58049 ± 0.03835	0.001
ACR-L	0.45650 ± 0.03309	0.42433 ± 0.03126	<0.001
ACR-R	0.47408 ± 0.03475	0.44139 ± 0.03467	0.001
SCR-L	0.49535 ± 0.02973	0.47041 ± 0.02517	0.001
SCR-R	0.49928 ± 0.02667	0.47364 ± 0.02556	0.001
PCR-L	0.42394 ± 0.02883	0.40000 ± 0.02896	0.003
PCR-R	0.44081 ± 0.02921	0.41456 ± 0.02912	0.001
PTR-L	0.55988 ± 0.03049	0.52517 ± 0.04548	0.001
PTR-R	0.59368 ± 0.04107	0.55587 ± 0.04868	0.003
IFO-R	0.47807 ± 0.02621	0.44830 ± 0.03220	<0.001
CGC-L	0.46867 ± 0.03465	0.43867 ± 0.03875	0.003
CGC-R	0.42969 ± 0.02742	0.40396 ± 0.02929	0.001
RLIC-R	0.53816 ± 0.02718	0.51165 ± 0.03047	0.001
SS-R	0.48288 ± 0.03446	0.45018 ± 0.03014	<0.001
EC-R	0.39192 ± 0.02754	0.36718 ± 0.02313	0.001

*GCC, genu of corpus callosum; BCC, body of corpus callosum; SCC, splenium of corpus callosum; ACR, anterior corona radiata; SCR, superior corona radiata; PCR, posterior corona radiata; PTR, posterior thalamic radiation; IFO, inferior fronto-occipital fasciculus; CGC, cingulate gyrus; RLIC, retrolenticular part of internal capsule; SS, sagittal stratum; EC, external capsule; L, left; R, right*.

#### Correlation Analysis in HIV+/Syphilis+

**Figure 4:**
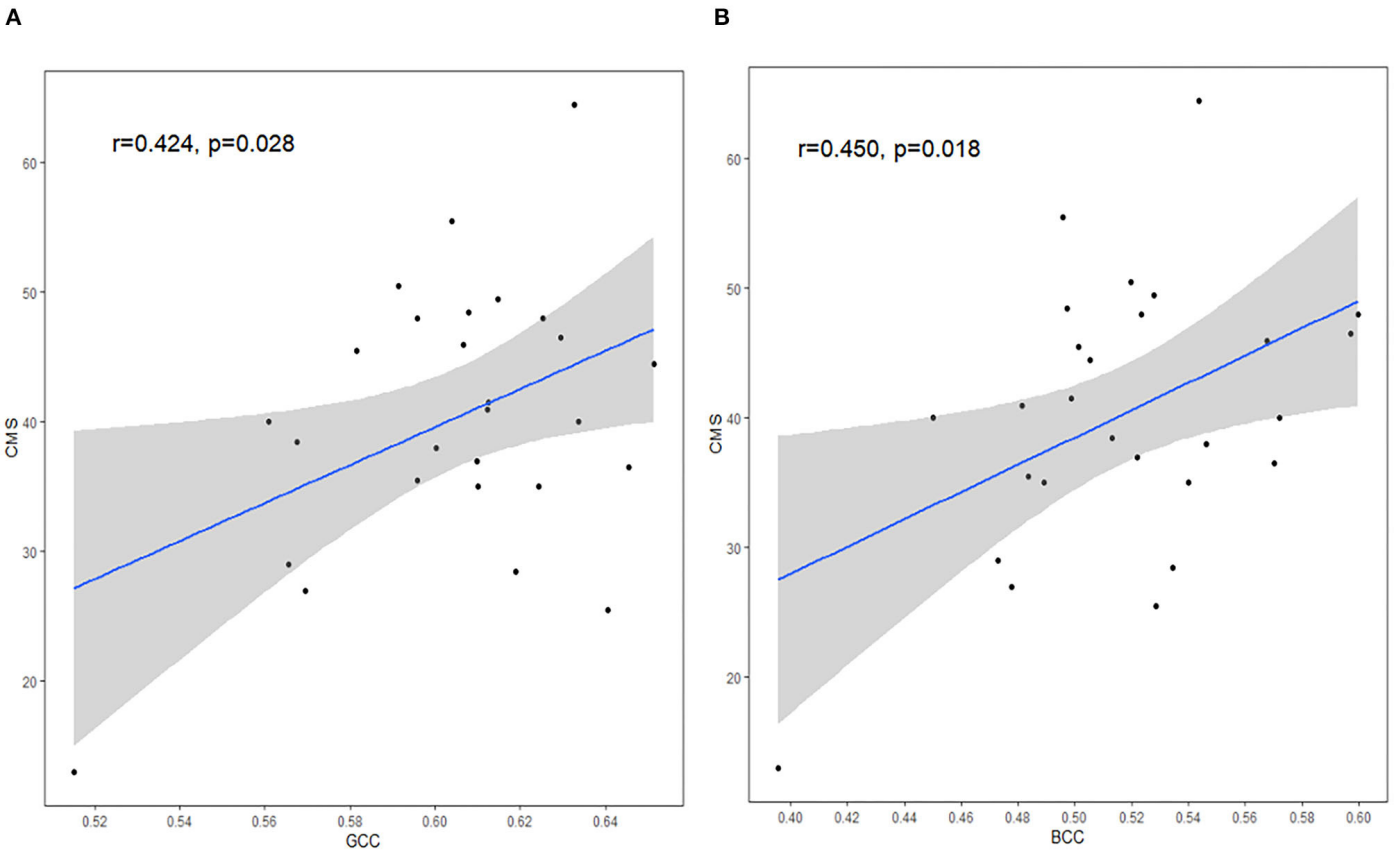
The correlation between DTI measures and CMS. FA in GCC **(A)** and FA in BCC **(B)** were positively correlated with CMS scores. GCC, genu of corpus callosum; BCC, body of corpus callosum; CMS, complex motor skills. The shaded area shows its 95% confidence interval. Significance level, *p* <.05.

Correlation analysis showed that there was a positive correlation between FA and cognitive tests, including CMS and GCC (*r* = 0.424, *p* = 0.028), CMS and BCC (*r* = 0.450, *p* = 0.018) (Figure 4). No significant correlations were found in any clinical data and cognitive tests (all *p* > 0.05, Pearson correlation). However, after Bonferroni correction for multiple comparisons, the findings were not significant.

## Discussion

We investigated the effect of syphilis infection on WM in HIV-infected patients using TBSS. Our study showed that the abnormal areas of WM found only FA and RD among the three groups. Compared with HC, there were no significant differences in HIV+/syphilis–, lower FA in BCC, SCC, GCC, the bilateral ACR, SCR, PCR, and PTR, higher RD in BCC and SCC was found in HIV+/syphilis+. Compared with HIV+/syphilis–, lower FA was found in BCC, SCC, GCC, the bilateral ACR, SCR, PCR, PTR, CGC, the right IFO, RLIC, SS, and EC in HIV+/syphilis+. Additionally, patients in the HIV+/syphilis+ group had a lower score in CMS. Correlation analysis uncorrected for multiple comparisons showed a positive correlation between FA in GCC and CMS, FA in BCC, and CMS in HIV+/syphilis+.

No significant difference was found between HC and HIV+/syphilis– group; the following related factors may be responsible for this result. Firstly, the median duration of infection time in this group was relatively shorter. During primary HIV infection (defined as <1 year after infection), the microstructure of WM was relatively intact; no abnormality was found in DTI measures (25). A study showed that the HIV subjects with average duration of 163 months had decreased FA only in the parietal lobe and increased MD in the frontal lobe (17). The average duration of the subjects in this group was just 21 months, which was inadequate to damage WM. Secondly, 79.3% (23/29) of the subjects received HAART, which can decrease brain viral levels, reduce, and even partially reverse alterations for neuronal injury (26). Thus, the damage in our group may be relatively milder and insufficient to contribute to WM changes.

Furthermore, lower FA and higher RD were found around the lateral ventricle in HIV+/syphilis+ compared with HC. The areas of higher RD were limited to the CC, and areas of lower FA extended to the areas adjacent to CC. Two possible reasons may be contributed to this result. Firstly, HIV can damage the microstructure of WM in HIV-infected individuals and is more sensitive to WM fibers around the lateral ventricle, such as CC, PCR, PTR, SCR, and ACR (27–29), but the exact reason is unclear; one reasonable explanation is that the virus can be present in the lateral ventricle, and, when the blood CSF barrier is broken, the virus can invade adjacent brain tissue (30). Secondly, the patients with HIV and syphilis co-infections can have a higher CSF HIV viral load (15); the CSF HIVRNA was positively correlated with the levels of various inflammatory cytokines (31); thus, a higher CSF HIV viral load may indicate a more active intracranial immune activation. HAART introduction can result in reducing CSF HIV viral load, but no response to immune activation (32). Thus, it is plausible that additive or synergistic effect on damage of WM in patients with HIV. It should be noted that a previous study showed the periventricular areas such as CC could be damaged without comorbidities in patients with HIV (29); in this study, however, all the patients had at least 5 years of HIV infection with median disease duration of 13 years. It is reasonable to assume that HIV infection without comorbidities will eventually damage the structures around the lateral ventricle over time. With the additive or synergistic effect of syphilis, the progress was accelerated. Interestingly, compared with HIV+/syphilis-, HIV+/syphilis+ exhibited a wide range of lower FA, especially in the distribution territory of middle cerebral artery without any change in RD. The potential treponema pallidum may involve small and medium blood vessels in CNS and then leading to endothelial damage and breakdown of BBB, especially the branches of middle cerebral artery, contributing to neural inflammation (33). However, only changes of FA were found, indicating that the damage, which may be a combination of pathological factors, demyelination, and axonal injury caused by syphilis in patients with HIV, may be inconspicuous. This result further illuminated that treponema pallidum may indirectly promote the destruction of brain tissue.

The exact underlying mechanisms for the abnormal FA and RD are unknown. Many factors can influence diffusion anisotropy, including the integrity of axonal membranes, pathways of neuronal fiber bundles, diameter of neuraxon, whether the neuraxon is tightly packed, the thickness of myelin sheath, the integrity of myelin sheath, microtubules, and fast axonal transport structures other than the axonal fibers. The primary factor is the integrity of axonal membranes (14, 34). In patients with HIV, beta-amyloid precursor protein (beta-APP), which symbolizes impaired axonal flow, were found in both supercifical and deep WM, suggesting widespread axonal injury (35). Myelin loss and antibodies against myelin basic protein were also found in patients with HIV (36). Previous DTI studies also suggested that axonal injury and myelin loss occurred in the patients with HIV (19). Furthermore, reactive astrocytosis and microglial proliferation were more frequent and severe in the patients with HIV (37). The combined effect of the above factors can have an impact on FA. Our study showed, compared with HC, higher RD was concentrated in BCC and SCC in the HIV+/syphilis+ group, suggesting that the diffusion rate in the perpendicular direction increased in CC, which may mainly result from loss of myelin. A wider lower FA was found in the HIV+/syphilis+ group; the reasons may be complicated. Although many previous DTI studies on HIV-infected patients have shown demyelination and axonal damage in WM, RD may be the predominant factor contributing to the changes of FA (29, 38). In our study, loss of myelin and axonal damage may have a relatively smaller effect on this due to syphilitic co-infection, and it is possible that, due to its proximity to the lateral ventricle, the CC was more severely damaged in myelin sheath. Compared with HIV+/syphilis–, HIV+/syphilis+ exhibited a widespread range of lower FA. This further suggested that the effects of syphilis co-infection on the patients with HIV were not limited to demyelination and axonal damage, whether the main influencing factor, which is the integrity of axonal membranes, and other factors such as damage of the collagenous perivascular fibrous alae (34), activation of innate immunity, and disruption of trophic coupling between vascular and brain cells have changed still remains unknown. The experiment needs to be replicated in larger sample to investigate the underlying cause of syphilis.

Although the mechanism of additive or synergistic effect of syphilis remains unknown, our results have implications for a clinic. HAART, which can decrease morbidity and mortality of AIDS but not cure HIV, is the main treatment for patients with HIV; currently, benzathine penicillin G remains the treatment of choice for syphilis (39). The therapy of syphilis in patients with or without HIV infection is identical; however, the risk of treatment failure is higher in patients with HIV infection (40). Those with treatment failure (<4-fold decline in titer) need retreatment, and follow-up serologies should be obtained every 3 months for at least 2 years; this may be inconvenient for patients (5, 40). Therefore, combined with the results of our study, we believe that, for the HIV-infected patients, good habits should be maintained to avoid syphilis infection.

In addition, there was a positive correlation between CMS and FA measures in GCC, and CMS and FA measures in BCC in the HIV+/syphilis+ group without multiple comparisons correction, the result indicated that there may be a relationship between imaging changes and cognitive function. The CC plays a role in transferring information between cerebral hemispheres. GCC mainly consists of frontal callosal fibers. Primary motor areas were found in the frontal lobe, including the anterior central gyrus, supplementary motor area (SMA), pre-SMA, and other areas related to voluntary movement. These areas have been shown to be involved in executive function, and complex movement. BCC contains auditory fibers, motor cortex fibers, somatosensory fibers, and parts of temporoparietal fibers. SCC contains visual callosal fibers (41). Injury to CC may affect visuospatial information transfer, motor skills, behavior, and consciousness (42). In Alzheimer's disease (AD), FA in GCC can significantly predict cognitive decline even after accounting for AD biomarkers (43). In traumatic brain injury, consciousness levels correlated strongly with a reduction of FA in CC (44). In the HIV group, areas around the lateral ventricle are easily involved; supporting this idea, previous studies found FA in CC was significantly correlated with motor speed deficits and visual memory deficits (42). Changes of GCC may contribute to cognitive deficits (17). In this study, we used Grooved Pegboard Test to evaluate CMS. Grooved Pegboard Test, which needs different parts of brain to coordinate, can assess manual dexterity, rapid visual-motor coordination, and psychomotor speed of patients (45). CC contains different types of fibers; thus, it is understandable that BCC and GCC showed correlation with CMS. Our result was basically consistent with previous studies (17, 46).

Our study has some limitations. Firstly, the limited sample size may result in lower power. Secondly, the limited diffusion MRI acquisition protocol may be only used for preliminary analysis of TBSS. To further explore, parameters need to be optimized. Thirdly, our study includes only male gender, which may prevent the results applicable to all populations or female patients.

## Conclusions

In conclusion, patients with HIV+/syphilis+ had a more serious damage of WM, which indicated that syphilis and HIV co-infection can have an additive or synergistic effect on the brain WM. HIV-infected patients without syphilis should be actively treated to avoid syphilis infection.

## Data Availability Statement

The raw data supporting the conclusions of this article will be made available by the corresponding author Hong-Jun Li on reasonable request.

## Ethics Statement

The studies involving human participants were reviewed and approved by the Ethics Committee of Beijing Youan Hospital, Capital Medical University. The patients/participants provided their written informed consent to participate in this study.

## Author Contributions

YQ and R-LL: research design, writing—original draft, writing—review, and editing. WW, Y-YW, J-JL, and XL: data collection. X-ZL, X-DZ, WY, and BR: data analysis. Y-FG, BR, and H-JL: writing—review and editing. All authors contributed to the article and approved the submitted version.

## Funding

This work was supported by National Key R&D Program of China (No. 2019YFE0121400), National Natural Science Foundation of China (Nos. 81771806, 61936013, and 81701679) supported the research design and collection, National Natural Science Foundation of China (No. 81771819) supported the data processing and analysis, Beijing Excellent Talent Plan (No. 2018000021469G290), and Natural Science Foundation of Tianjin (No. 19JCQNJC09800) supported the writing and revising the manuscript.

## Conflict of Interest

The authors declare that the research was conducted in the absence of any commercial or financial relationships that could be construed as a potential conflict of interest.

## Publisher's Note

All claims expressed in this article are solely those of the authors and do not necessarily represent those of their affiliated organizations, or those of the publisher, the editors and the reviewers. Any product that may be evaluated in this article, or claim that may be made by its manufacturer, is not guaranteed or endorsed by the publisher.
